# Collective Cognition on Global Density in Dynamic Swarm

**DOI:** 10.3390/s23104648

**Published:** 2023-05-11

**Authors:** Phan Gia Luan, Nguyen Truong Thinh

**Affiliations:** Institute of Intelligent and Interactive Technologies, University of Economics Ho Chi Minh City, Ho Chi Minh City 700000, Vietnam; luanpg@ueh.edu.vn

**Keywords:** swarm robots, collective cognition, hard-core point process, collective decision making

## Abstract

Swarm density plays a key role in the performance of a robot swarm, which can be averagely measured by swarm size and the area of a workspace. In some scenarios, the swarm workspace may not be fully or partially observable, or the swarm size may decrease over time due to out-of-battery or faulty individuals during operation. This can result in the average swarm density over the whole workspace being unable to be measured or changed in real-time. The swarm performance may not be optimal due to unknown swarm density. If the swarm density is too low, inter-robot communication will rarely be established, and robot swarm cooperation will not be effective. Meanwhile, a densely-packed swarm compels robots to permanently solve collision avoidance issues rather than performing the main task. To address this issue, in this work, the distributed algorithm for collective cognition on the average global density is proposed. The main idea of the proposed algorithm is to help the swarm make a collective decision on whether the current global density is larger, smaller or approximately equal to the desired density. During the estimation process, the swarm size adjustment is acceptable for the proposed method in order to reach the desired swarm density.

## 1. Introduction

Swarm robotics is a sub-field of multi-agent robotics that employs distributed controllers, where inter-robot local communication is the key component. Due to the use of distributed controllers in a self-organized manner, swarm robot systems are expected to have three main advantages: flexibility, scalability and robustness. However, to design self-organized mechanisms is challenging, as desired behavior at the macroscopic level must be accomplished based on a set of behaviors programmed in each individual robot. Many task-based controllers have been designed for self-organized swarm robot systems such as collective transporting [1,2,3], collective foraging [4,5], collective decision-making [6,7] and collective exploration [8]. These controllers primarily focus on the scalability of swarm systems, in which changes to the swarm size within a reasonable range do not dramatically affect the performance of the swarm robots.

The average performance of a robot swarm which uses previous controllers actually depends on the swarm density, and it only depends on the swarm size if the operating area is kept constant. The analyses in previous works [1,3,9] has shown that the performance of swarms can be optimized by determining a suitable swarm density. In high-density environments, robots are compelled to put in more effort and attention to address inter-robot collision avoidance tasks rather than concentrating on their core responsibility, which lowers the swarm’s overall performance. If the robot density is raised much further, performance will likely decrease to negligible levels due to interference halting almost all motion. However, when there are too few robots present in a large area, robot collaborations are noticeably ineffective, and the same outcome could be observed. In some cases, low-density swarms can have more impact than high density situations, as observed in self-organized aggregation processes [10,11]. Hence, in order to optimize the performance of the swarm, the swarm density must be predetermined.

Even if the theoretical optimal swarm density is determined, the performance of the swarm may not reach its maximum value due to unmeasurable swarm density in real-world situations. Generally, swarm density is defined as the number of robots occupying a unit area [9]. However, in many cases, swarm density is unknown due to the lack of knowledge about the workspace. Even if the workspace is fully determined, the varying swarm size is also a significant issue in measuring swarm density. Faulty or out-of-battery robots are factors that make swarm density vary with respect to time. With large swarm sizes, more robots may break, get lost or may intentionally be removed, causing the swarm density to decrease at runtime. Real-time changes in density have an impact on the swarm performance, and if there is no compatible swarm size change, the performance of the swarm will be greatly reduced.

There are many previous studies that can help swarm robots collectively determine swarm density. Sheng Zhao et al., proposed the Smoothed Particle Hydrodynamic (SPH) model which uses the weighted sum of distances to its nearby robots within a certain range as the robot density [12]. This information is locally utilized by a robot which does not imply any collective information. Inter-robot collisions are a simple and effective way to derive swarm density. Many social insect colonies, according to entomological studies, can monitor density through inter-individual collision in order to manage interference with decentralized mechanisms [13,14]. Siddharth, et al., who were inspired by these species, created a model that was used to compute the swarm density using the frequency of inter-robot interactions [15]. The approach has been used for certain behaviors such as group gathering [16] and task allocation [17].

This work introduces an approach that enables swarms of robots to collectively determine whether their current swarm density is greater, smaller or comparable to a given desired swarm density. The fundamental idea of the approach is to enable the robots to estimate the swarm density collectively by evaluating how frequently they encounter their neighbors, which allows the swarm as a whole to make decisions. The relationship between encounter rate and swarm density is modeled based on the nearest neighbor distance distribution in the hard-core point process introduced in [18]. To ensure the swarm has shared information to make the same decision, the simple distributed average consensus strategy is proposed. The proposed process addresses four main challenges of designing collective swarm density cognition: (1) swarm density is a global feature that can only be efficiently detected collectively by applying the distributed average consensus technique; (2) we face a tradeoff between filtering noise and reacting quickly to changes; (3) the swarm density estimator only depends on swarm size and environment area and does not require knowledge of additional parameters such as environment shape, robot speed or robot kinematics, as long as the robot motion is ergodic; (4) the method should be able to assess changes in swarm density over time to reach optimal density. The proposed method is verified by numerous simulations and is also deployed on a team of real robots. By analyzing the results, the collective swarm density cognition method has an applicable response to the change of swarm density with an acceptable error when a significant number of changes occurs in the swarm density.

## 2. Task Formulation

Consider a finite swarm of identical robots, denoted by $S\left(t\right)=\left\{R_{1},R_{2},\ldots \right\}$, which are randomly distributed in a compact, connected planar domain $D\subset {\mathbb{R}}^{2}$ where $R_{i}\in S\left(t\right)$ denotes the i^th^ robot. The swarm size can be varied during the operation of the swarm, and it is denoted as $N\left(t\right)$. Let $r_{r}$ denote the robot footprints’ actual physical radius. Each robot is equipped with sensors that helps it perceive the presence of obstacles and other robots with a detecting skirt of the total diameter 2$r_{s}$ around it. The same range is applied to the robots’ communication system, which enables robots to exchange explicit information with their neighbors. Additionally, it is expected that any D can fit all $N\left(t\right),\forall t\geq 0$ robots without encountering any challenging geometric packing issues. Let $\lambda \left(t\right)$ denote the population density of swarm $S\left(t\right)$ over domain D which can be defined as Equation (1).

(1) $$\lambda \left(t\right)=\frac{N\left(t\right)}{\left|D\right|}$$

where $\left|\cdot \right|$ denotes the set area operator. Following the preliminary introduction, it is assumed that the swarm operates in a fully or partially unknown domain, and the size of the swarm may vary. Hence, λ is a time-varying variable that can only be estimated. However, in this study, we do not attempt to propose the method used to estimate λ. The key issue we will tackle is how to answer the question of whether a swarm’s actual swarm density surpasses, falls short of or is roughly equal to a given desired swarm density, $\lambda_{d}$, by introducing a distributed algorithm that aids swarms. Furthermore, we assume that robots in a swarm do not have any prior knowledge of the size of the workspace, and the decision of robots in the swarm is based solely on the information related to the inter-robot encounter rate.

However, the rate of encounters experienced by the robots will depend on their motion in the domain. We initially restrict the motion characteristics of the robots in their workspace in order to make specific claims about inter-robot encounters. Let $x_{i}\left(t\right)\in D$ be the position of i^th^ robot at time t. Then, for any given subset U⊆D, the average time spent by the robot in that set should be equal to the ratio defined in Equation (2). If the Equation (2) is held in the swarm, then we can state that the motion pattern of robots in the swarm is ergodic.

(2) $$\underset{T\to \infty }{\lim}\int_{t=0}^{T}\frac{1}{T}\sum_{i=1}^{N}1\left(x_{i}\left(t\right)\in U\right)dt=\frac{\left|U\right|}{\left|D\right|}$$

The developed encounter model is applicable as long as the ergodicity assumptions are satisfied, which enables us to make generalizations about the types of motion patterns that arise from the task’s execution. The consistently ergodic trajectories of the robots imply that their density over the domain D at any given instant is also uniform. In our study, the motion of robots in a swarm can be summarized by the following steps: (1) a robot moves forward; (2) if a robot comes close to the workspace boundaries or collides with other robots, it turns in a randomly selected direction and reverts back to step (1). As long as the ergodicity assumption is still valid, the other motion models can be used even when robots are performing other tasks while executing the suggested mechanism.

## 3. Encounter Rate

In the first section, we stated that the robots performing the collective task in a distributed manner are equipped with sensors that can help the robot detect the presence of its neighbor within the range of $r_{s}$. We first denote that $F_{i}\left(t\right)\subset S\left(t\right)$ is the set of robots in range of $r_{s}$ within $R_{i}$, called neighbor set of $R_{i}$, in which if $R_{j}\in F_{i}\left(t\right)$, then $\left\|x_{i}\left(t\right)-x_{j}\left(t\right)\right\|<r_{r}+r_{s}$ must be satisfied. Hence, we define a robot as having an “encounter” when a neighbor set of given robots is not empty, $F_{i}\left(t\right)\neq \emptyset$. Based on the given motion pattern of robots in the swarm, upon colliding with another robot, each robot executes a collision avoidance maneuver to prevent the collision and continue along its trajectory. Consequently, the presence of the complement state of “encounter” is called “disjoin” where no robots are present in the sensory range of a given robot or $F_{i}\left(t\right)=\emptyset$.

We first need to construct a model for how frequently encounters happen among the robots, or the encounter rate, in order to be able to use encounter information to estimate the swarm density. The robot’s encounter rate can be calculated from its time in the encounter and disjoin states. However, the durations indicating how long a robot stays in an encounter or disjoin state are stochastic variables due to the randomness of the motion model. Obviously, these random variables are dependent on swarm density as well as on different distribution parameters with regard to how quickly the task of avoiding collisions may be solved and the robot speed. These factors can be modeled, but they can also complicate the estimation process and reduce its generality. Firstly, the collision avoidance strategy and time spent on different locomotion methods—for example, differential-drive robots and omnidirectional robots that share physical parameters—are significantly different. Furthermore, although robot density is maintained as constant, the ratio of encounter duration to total process duration varies during the process due to the varying speed of robots. To ensure the generalizability of the proposed method, the measurement of travelled distance in which these states are occupied are implemented instead of measuring in terms of time. From now on, s will be used as the discrete index parameter indicating the travelled distance of a given robot. We should note that the travelling distances among robots in a swarm are not the same with respect to time.

Hence, now, the states of each robot in the swarm can be modelled as a continuous stochastic chain $\left\{X\left(s\right):s\geq 0\right\}$ defined by index parameter s and state space $\left\{0,1\right\}$ where 0 denotes the “disjoin state” and 1 denotes the “encounter state”. Due to the exponential decrease in probability of travelling distance for a single encounter event and single disjoin event, and their independence from each other, this process can be considered as a continuous Markov process with an index parameter in terms of the travelled distance. The travelled distance on the single encounter event and single disjoin event can be modelled as the exponential distribution with rate parameters $\lambda_{e}$ and $\lambda_{d}$, respectively. Let $\pi_{e}$ be the average encounter rate of robots in the swarm, and $\pi_{e}\left(t\right)$ can be defined as a stationary probability of the encounter state at time t.

(3) $$\pi_{e}\left(t\right)=\frac{\lambda_{d}\left(t\right)}{\lambda_{e}\left(t\right)+\lambda_{d}\left(t\right)}$$

However, because $\lambda_{e}$ and $\lambda_{d}$ are unknown variables and depend on the number of robots in the swarm, it is possible to estimate $\pi_{e}$ solely by using estimators for $\lambda_{e}$ and $\lambda_{d}$. Let ${\hat{\pi }}_{e,i}$ be the encounter rate estimator of $R_{i}$, which can be defined as Equation (4) by applying the moment method.

(4) $${\hat{\pi }}_{e,i}\left(t\right)=\frac{{\hat{\lambda }}_{d,i}\left(t\right)}{{\hat{\lambda }}_{e,i}\left(t\right)+{\hat{\lambda }}_{d,i}\left(t\right)}$$

where ${\hat{\lambda }}_{e,i}$ and ${\hat{\lambda }}_{d,i}$ are the estimators of the inverse of the mean of travelled distance on the encounter state and disjoin state of the given continuous Markov process estimated by $R_{i}$. The estimation procedure can be carried out by using the maximum likelihood estimation method on two sets of random samples. The first set consists of the length of travelled distance a robot spends in the encounter state, while the second set consists of the sum of its travelled distance spent in both states. k represents the size of each set. The value of k can be viewed as the number of cycles of the Markov process that a particular robot has gone through. However, directly determining the value of ${\hat{\pi }}_{e,i}$ can be very complicated. To ease this problem, the auxiliary estimator $z_{i}$ is employed which is defined in Equation (5).

(5) $$z_{i}\left(t\right)=\frac{\sum_{j=1}^{k_{i}}s_{e,i,j}}{\sum_{j=1}^{k_{i}}\left(s_{e,i,j}+s_{d,i,j}\right)}$$

where $s_{e,i,j}$ and $s_{d,i,j}$ are the travelled distance of the j^th^ encounter event and j^th^ disjoin event measured by $R_{i}$. Both $s_{e,i,j}$ and $s_{d,i,j}$ are random variables which have a probability density function represented by an exponential model. Hence, if the change of average global density is slow enough, then we can approximate that $\sum_{j=1}^{k_{i}}s_{e,i,j}\sim \Gamma \left(k,k\lambda_{e}\right)$ and $\sum_{j=1}^{k_{i}}\left(s_{e,i,j}+s_{d,i,j}\right)\sim \Gamma \left(k,k\lambda_{c}\right)$ where $\Gamma \left(a,b\right)$ represents the gamma distribution parameterized in term of shape parameter a and an inverse scale parameter b; $\lambda_{c}$ can be defined as Equation (6).

(6) $$\lambda_{c}=\frac{\lambda_{d}+\lambda_{e}}{\lambda_{d}\lambda_{e}}$$

According to [19], the probability distribution of $z_{i}\left(t\right)$ can be approximate to the generic beta model of the second kind, i.e., $z_{i}\sim GB2\left(k,k,1,\frac{\lambda_{c}}{\lambda_{e}}\right)$ where the first three parameters are shape parameters and $\lambda_{c}/\lambda_{e}$ is the scale parameter. The distribution of $z_{i}$ is defined in terms of the probability density function (Equation (7)).

(7) $$f_{z_{i}}\left(z\right)=\frac{\lambda_{e}\Gamma \left(2k\right)}{\lambda_{c}\Gamma^{2}\left(k\right)}{\left(\frac{z\lambda_{e}}{\lambda_{c}}\right)}^{k-1}{\left(1+\frac{z\lambda_{e}}{\lambda_{c}}\right)}^{-2k}$$

In which $\Gamma \left(.\right)$ is the gamma function of a given parameter. Note that if k<1, then the mean of $z_{i}$ is infinite and if k<2, then the variance of $z_{i}$ is not defined. Hence, to be able to determine these values, k should be chosen as greater than 2. We assume that k>2; then, the mean and variance of $z_{i}$ are defined in Equations (8) and (9), respectively.

(8) $$E\left(z_{i}\right)=\frac{\lambda_{c}}{\lambda_{e}}\frac{k}{k-1}$$

(9) $$\mathrm{var}\left(z_{i}\right)={\left(\frac{\lambda_{c}}{\lambda_{e}}\right)}^{2}\frac{k\left(2k-1\right)}{\left(k-2\right){\left(k-1\right)}^{2}}$$

According to Equation (8), ${\hat{\pi }}_{e,i}$ can be estimated by $E\left(z_{i}\right)$ via Equation (10).

(10) $${\hat{\pi }}_{e,i}=E\left(z_{i}\right)\frac{k-1}{k}$$

The choice of k affects both the correctness of the estimated value ${\hat{\pi }}_{e,i}$ and the sensitivity of robots to the change of average global density. If k is increased, then the range of possible values of ${\hat{\pi }}_{e,i}$ is narrowed down to $E\left({\hat{\pi }}_{e,i}\right)$, but in order to notice changes in the swarm density, the robot needs to take a longer time. Another issue is that the time required to finish k samples depends on the swarm density when the number of samples k is fixed, rather than using a fixed sampling time. In other words, if the swarm density approaches 0, collecting k samples could take an infinitely long time. Therefore, the maximum sample period in terms of travelled distance $S_{s}$ is provided as a solution to this issue. During the sampling time, if any measured event duration $s_{e,i,j}$ or $s_{d,i,j}$ of $R_{i}$ exceeds $S_{s}$, then the estimating process will be terminated and considered as completed. In this case, if $\sum_{j=1}^{k_{i}}s_{e,i,j}<\sum_{j=1}^{k_{i}}s_{d,i,j}$, then ${\hat{\pi }}_{e,i}:=0$ and vice versa, ${\hat{\pi }}_{e,i}:=1$. The selection of $S_{s}$ affects the minimum and maximum measurable value of ${\hat{\pi }}_{e,i}$ and the sensitivity of the robot to the change of swarm density. In the next section, we will estimate the likelihood of a robot encountering another robot at a specific time ${\hat{\pi }}_{e,i}\left(t\right)$ as a function of the partial distribution of robots throughout the relevant domain.

## 4. Nearest Neighbor Distance Distribution

Under the definition of an encounter state, an encounter is said to occur if the given robot, whose position is denoted as $x_{i}$, has at least one neighbor that is equivalent to the distance from $x_{i}$ to the nearest neighbor of a given robot, whose position is denoted as $x_{j}$ and is less than $r_{r}+r_{s}$. Hence, the encounter rate or the ratio that $R_{i}$ experienced the encounter state in terms of travelled distance can also be determined by the probability that the random variable denoting the distance between $R_{i}$ and its nearest neighbor $R_{j}$ is less than $r_{r}+r_{s}$. Due to the time invariance of the encounter rate, this kind of probability only depends on the partial distribution of swarm robots over the domain. Hence, the patterns of geometrical swarm over the interested domain must be constructed first.

Point processes are most commonly used as a mechanism to interpret patterns of geometrical objects—for example, the position of swarm robot in our case. In this study, we describe the spatial distribution of the swarm of robots in a certain workspace using a homogeneous point process. Each robot is represented by a point in the point process, which also indicates where it is in the workspace. The Poisson process is the most basic type of point process. The selection of completely independent random points without observing the minimum distance is modelled by the Poisson process. However, the Poisson process is not suitable for modelling the position distribution of robots since it assumes that the locations of the robots are independent and uniformly distributed, which does not take into account the physical footprint of the robots or the fact that they cannot overlap.

The purely random selection of positions without observing a minimum distance is modelled by the Poisson process. Meanwhile, a hard-core process is a stochastic point process in which successive events maintain a specified minimum distance from one another. It is suitable for modelling the partial distribution of robots in a swarm. From the perspective of stochastic geometry, there exist n-dimensional point fields generated by hard-core processes using the center of n-dimensional balls that do not overlap and have a given diameter. Depending on the way in which the points are generated, different hard-core processes with different properties can be described.

**Figure 1:**
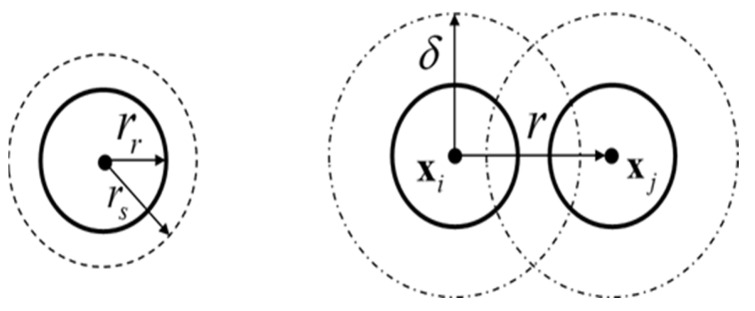
(**Left**): the geometric parameters of the robot; (**right**): the geometric parameters between robots.

Performing a dependent-thinning approach on an underlying “parent” spatial Poisson process, where points are gradually deleted until the appropriate hard-core requirements are satisfied by all the remaining points, is a typical technique for creating hard-core point processes. In this study, we focus on MHC type-II, fully detailed in [20]. To construct an MHC type-II process, we start with a real number δ>0 and a parent stationary parent Poisson process denoted by $\Phi_{p}$, having a homogeneous intensity $\lambda_{p}$ (points per unit area) and then for each point $x\in \Phi_{p}$, a uniformly distributed random mark $Μ\left(x\right)∼U\left(0,1\right)$ is assigned. If the mark of point $x\in \Phi_{p}$ is not the lowest relative to all other points in a ball with radius δ, $b\left(x,\delta \right)$, around it, that point will be marked for removal. Only after this test is done for all points in $\Phi_{p}$, the points that were flagged-for-removal are removed. The retained points constitute the resulting MHC process $\Phi_{hc}$. The distribution of robots over D can be modelled by using $\Phi_{hc}$ with parent $\Phi_{p}$ over D. Due to the removal of some points in the origin process, the point density of $\Phi_{hc}$ is not larger than the one in $\Phi_{p}$. Let $\lambda_{hc}$ denote the hard-core point process density; the relation between $\lambda_{hc}$ and $\lambda_{p}$ can be formulated as the following:

(11) $$\lambda_{p}\pi \delta^{2}=\ln\left(\frac{1}{1-\lambda_{hc}\pi \delta^{2}}\right)$$

where δ is also called the hard-core distance of $\Phi_{hc}$ which is illustrated in Figure 1. In our case, δ can also be interpreted as the minimum distance between two robots, i.e., $\delta =2r_{r}$. Furthermore, the distribution of the point in $\Phi_{hc}$ over D represents the partial distribution of the swarm robot over the same domain, resulting in $\lambda_{hc}=\lambda$. According to the proposed motion model, in this study, the MHC process $\Phi_{hc}$ with population density λ is used to describe the partial distribution of the swarm robot over D. Based on the constructed partial distribution of the swarm robots, the probability of experiencing an encounter state of $R_{i}$ will be calculated.

**Figure 2:**
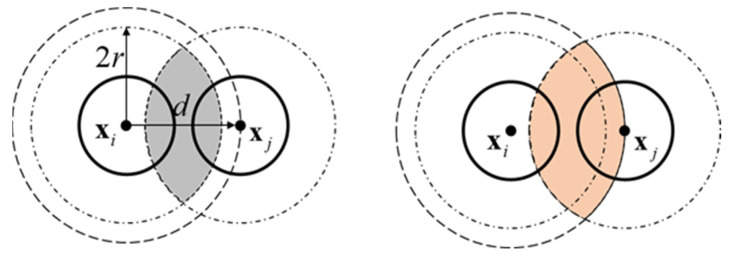
(**Left**): the black shaded region is the lens formed by the intersection of $b(x_{i},\delta )$ and $b(x_{j},\delta )$; (**right**): the orange shaded region is the lens formed by the intersection of $b\left(x_{i},r\right)$ and $b\left(x_{j},\delta \right)$.

To construct the nearest neighbor distance model, a geometric function must be determined first. According to Figure 1, let r be the distance between $R_{i}$ and its nearest neighbor; $l_{1}\left(r,\delta \right)$ be the area of the symmetrical lens formed by the intersection of $b\left(x_{i},\delta \right)$ and $b\left(x_{j},\delta \right)$; and $l_{2}\left(r,\delta \right)$ be the area of the asymmetrical lens formed by the intersection of $b\left(x_{i},r\right)$ and $b\left(x_{j},\delta \right)$. $l_{1}\left(r,\delta \right)$ and $l_{2}\left(r,\delta \right)$ are illustrated in Figure 2 and can be defined as in Equations (12) and (13), respectively.

(12) $$l_{1}\left(r,\delta \right)=\left\{\begin{array}{cc} 2\delta^{2}\cos^{-1}\left(\frac{r}{2\delta }\right)-\frac{1}{2}r\sqrt{4\delta^{2}-r^{2}}, & 0<r\leq 2\delta \\ 0, & r>2\delta \end{array}\right.$$

(13) $$l_{2}\left(r,\delta \right)=\left\{\begin{array}{cc} \pi r^{2}, & 0<r<\frac{\delta }{2}\\ r^{2}\cos^{-1}\left(1-\frac{\delta^{2}}{2r^{2}}\right)+\delta^{2}\cos^{-1}\left(\frac{\delta }{2r}\right)-\frac{1}{2}\delta \sqrt{4r^{2}-\delta^{2}}, & r\geq \frac{\delta }{2} \end{array}\right.$$

According to [18], the cumulative probability function of the distance between a given point and its neighbor, who is likewise provided by the MHC process $\Phi_{hc}$, can be approximated as Equation (14).

(14) $$F_{R}\left(R\right)=1-\exp\left(-\frac{2\pi \lambda_{p}^{2}}{\lambda_{hc}}\int_{\delta }^{R}r\kappa_{1}\left(r,\delta \right)dr\right)$$

where R is the random variable of nearest neighbor distance of a given point and $\kappa_{1}\left(r,\delta \right)$ can be expressed by Equation (15) with the components; $a=\lambda_{p}\pi \delta^{2}$, $b_{1}=\lambda_{p}l_{1}\left(r,\delta \right)$ and $b_{2}=\lambda_{p}l_{2}\left(r,\delta \right)$ are the expected numbers of the point occupying $b\left(x_{i},\delta \right)$, $l_{1}\left(r,\delta \right)$ and $l_{2}\left(r,\delta \right)$ in the parent Poisson process $\Phi_{p}$, respectively.

(15) $$\kappa_{1}\left(r,\delta \right)=\frac{1-\exp\left(-a\right)}{a^{2}-ab_{2}}+\frac{\exp\left(b_{2}-2a\right)-1}{2a^{2}+b_{2}^{2}-3ab_{2}}+\frac{\frac{1-\exp\left(-b_{1}+b_{2}-a\right)}{b_{1}-b_{2}+a}+\frac{\exp\left(b_{2}-2a\right)-1}{2a-b_{2}}}{\left(a-b_{1}\right)}$$

As stated, the likelihood that the random variable expressing the distance between the given robot and its nearest neighbor is smaller than $r_{r}+r_{s}$ can also be used to calculate the encounter rate or probability that the given robot experienced an encounter state in terms of travelled distance. Hence, according to (3) and (14), the relation between nearest neighbor distance distribution and encounter rate is determined as in Equation (16).

(16) $$\pi_{e}=F_{R}\left(r_{r}+r_{s}\right)$$

Based on Equation (16), if the desired swarm density $\lambda_{d}$ is given, the desired encounter rate can be defined. Let $\pi_{e,d}$ be the preassigned desired encounter rate for the swarm, and let $Q\in \left\{-1,0,1\right\}$ be the desired decision of the swarm, where the value of Q is determined based on the comparison between the current encounter rate and the desired encounter rate, as well as the threshold error σ.

(17) $$Q=\left\{\begin{array}{cc} -1 & \pi_{e}-\pi_{e,d}<\sigma \\ 0 & \left|\pi_{e}-\pi_{e,d}\right|\leq \sigma \\ 1 & \pi_{e}-\pi_{e,d}>\sigma \end{array}\right.$$

However, due to the randomness of encounter rate estimators, the values of $Q_{i}$, the decision of $R_{i}$ based on ${\hat{\pi }}_{e,i}$ instead of $\pi_{e}$, are different among swarm robots. This variation increases when $\pi_{e}\to \pi_{e,d}$ or σ→0. The technique that allows the entire swarm to make the same conclusion is introduced in the following section.

## 5. Collective Decision Making

According to Section 3, the estimated encounter rate ${\hat{\pi }}_{e,i}$ of $R_{i}$ can be determined by Equation (5). Once each robot has computed ${\hat{\pi }}_{e,i}$, they need to achieve consensus on the global value. However, the randomness of ${\hat{\pi }}_{e,i}$ prevents $Q_{i}$ from sharing the same value across the whole swarm. This problem falls into a broader class of distributed consensus or agreement problems in multi-agent coordination and wireless sensor networks [21]. These protocols allow a swarm to reach an agreement on a quantity of interest by exchanging information over the communication network. Consensus algorithms have the attractive property that, at termination, the computed value is available throughout the swarm, so a swarm user can query any robot and immediately receive a response, rather than waiting for the query and response to propagate to and from a fusion center. Additionally, there is no single-dead-point where the result of in-swarm computation can be biased due to undesired values from failed robots. However, previous consensus algorithms require a fixed network topology and multi-hop data transmission. In this section, we propose an implementation of the quasi-distributed average consensus algorithm that does not rely on multi-hop communication and can adapt to a dynamic swarm. The main idea of the proposed method is that each robot updates its value by a weighted sum of values from itself and its neighbor, i.e.,

(18) $$\pi_{e,i}\left(n_{i}+1\right)=\sum_{j=1}^{N}W_{ij}\left(n_{i}\right)\pi_{e,j}\left(n_{i}\right)$$

where $n_{i}$ is a discrete-time index taking values in the non-negative integers. In this section, we only consider ${\hat{\pi }}_{e,j}$ in the consensus process, and we use $n_{i}$ as its index value. Obviously, we have that ${\hat{\pi }}_{e,j}\left(0\right)={\hat{\pi }}_{e,j}$, where ${\hat{\pi }}_{e,j}$ represents the estimated encounter rate which is obtained by estimating the process. Here, $W_{ij}\left(n_{i}\right)$ is the linear weight on ${\hat{\pi }}_{e,j}\left(n_{i}\right)$ at $R_{i}$. Now, the question is how to choose the weight $W_{ij}\left(n_{i}\right)$ such that every ${\hat{\pi }}_{e,i}\left(n_{i}\right)$ converges to the average of their initial value, i.e.,

(19) $$\underset{n_{i}\to \infty }{\lim}{\hat{\pi }}_{e,i}\left(n_{i}\right)=N^{-1}\sum_{i=1}^{N}{\hat{\pi }}_{e,i}\left(0\right)$$

Equation (18) can be interpreted as a local average if the value of $W_{ij}\left(n_{i}\right)$ is chosen to follow the nearest neighbor rules proposed in [22]. This weight can simplify the communication method since it depends only on the value of the neighbor set and the number of neighbors.

(20) $$W_{ij}\left(n_{i}\right)=\left\{\begin{array}{cc} \frac{1}{1+\left|F_{i}\left(t_{i}\left(n_{i}\right)\right)\right|} & R_{j}\in F_{i}\left(t_{i}\left(n_{i}\right)\right)\;\mathrm{or}\;i=j\\ 0 & otherwise \end{array}\right.$$

in which $t_{i}\left(n_{i}\right)$ represents the time that $R_{i}$ determines $\pi_{e,i}\left(n_{i}+1\right)$. To simplify the notation, vector notation is used. Let ${\hat{\pi }}_{e}\left(m\right)={\left[{\hat{\pi }}_{e,1}\left(n_{1}\right),{\hat{\pi }}_{e,2}\left(n_{2}\right),\ldots {\hat{\pi }}_{e,N}\left(n_{N}\right)\right]}^{T}$ be the vector that represents the set of the estimated encounter rate of the swarm, in which m is a discrete-time index value. The value of m can be interpreted as the total number of non-simultaneous encounter rate updates. This way, the distributed averaging algorithm (13) can be written as Equation (21).

(21) $${\hat{\pi }}_{e}\left(m+1\right)=W\left(m\right){\hat{\pi }}_{e}\left(m\right)$$

where the weight matrix $W\left(m\right)\in {\mathbb{R}}^{NxN}$ is given by (20) and it satisfies the conditions required for the asymptotic average consensus $W\left(m\right)1=1$, $1^{T}W\left(m\right)=1^{T}$ and $\rho \left(W\left(m\right)-N^{-1}11^{T}\right)<1$, in which 1 stands for the vector of ones and $\rho \left(.\right)$ is the spectral radius of a given matrix. If Equation (16) is expanded by recursively replacing $\pi_{e,j}\left(t\right)$ by Equation (16), then the value of ${\hat{\pi }}_{e}\left(m+1\right)$ can be derived from ${\hat{\pi }}_{e}\left(0\right)$.

(22) $${\hat{\pi }}_{e}\left(m+1\right)=\left(\prod_{i=0}^{m}W\left(i\right)\right){\hat{\pi }}_{e}\left(0\right)$$

By applying the weight matrix with components defined in Equation (20) to Equation (22), Morse et al. [22] proved that every individual in the robot swarm will converge to the same value as in Equation (19), resulting in Equation (23).

(23) $$\underset{m\to \infty }{\lim}\prod_{i=0}^{m}W\left(i\right)=N^{-1}11^{T}$$

To make the proposed method able to be applied, the inter-robot communication mechanism must be clarified first. The first requirement of the communication mechanism is that each robot must have both broadcast and directional communication. To do that, infrared-based communication is employed [23]. Fortunately, this type of communication using an infrared medium can prevent most signals, and messages created by infrared transceivers can be modulated. The second requirement is bidirectional communication in which if a robot receives any messages from its neighbor, it has the responsibility to reply to the sender.

The consensus algorithm is asynchronously applied to the robot as soon as the robot acquires ${\hat{\pi }}_{e,i}\left(0\right)$. In this process, the robot will broadcast its current estimated encounter rate ${\hat{\pi }}_{e,i}\left(n_{i}\right)$ to its neighbors if it has no received messages for the duration $S_{q}$ in terms of travelling distance. According to the second requirement, all robots receiving that message will reply to their current estimated encounter rate first; then, it applies the recorded value to Equation (18). After receiving all replies, $R_{i}$ also applies all recorded values to Equation (18).

Equation (18) is the ideal result that can make a member of ${\hat{\pi }}_{e}\left(m\right)$ converge to $N^{-1}11^{T}{\hat{\pi }}_{e}\left(0\right)$; by this, the decisions of the whole swarm approach is totally collective. However, to help the swarm make the decision based on Equation (17), the sign to indicate the complete process must be pointed out. Let $range\left(a\right)$ denote the range of the arbitrary set of number a; the main purpose of this method is narrowing down $range\left({\hat{\pi }}_{e}\left(m\right)\right)$ to a value that can make the swarm have a collective sense about the estimated value. Let ϑ indicate the acceptable range of ${\hat{\pi }}_{e}$. Obviously, if $range\left({\hat{\pi }}_{e}\left(m\right)\right)\leq \vartheta$, then the condition in Equation (24) must be held.

(24) $$P\left(\left|{\hat{\pi }}_{e,i}\left(n_{i}\right)-{\hat{\pi }}_{e,j}\left(n_{j}\right)\right|\leq \vartheta \right)=1,\forall i,R_{j}\in F_{i}\left(t\left(n_{i}\right)\right)$$

Based on this condition, the consensus process of the swarm can be considered as finished. Let $S_{u}$ be the discrete sampling duration of $\left|{\hat{\pi }}_{e,i}\left(n_{i}\right)-{\hat{\pi }}_{e,j}\left(n_{j}\right)\right|$ in terms of the travelling distance of robots in the swarm. $S_{u}$ will start counting if the condition in Equation (24) is satisfied. During the travelled distance $S_{u}$, if the given condition is continuously satisfied, then the consensus process is considered as finished. At any time, if the above condition is not satisfied, $S_{u}$ will be reset and pause until Equation (24) is satisfied again. Therefore, $S_{u}$ should be chosen so as to achieve a trade-off between the steady-state of convergence and the ability of the robot to track changing densities. In case of ${\hat{\pi }}_{e,i}\left(0\right)=0$ or ${\hat{\pi }}_{e,i}\left(0\right)=1$, if the robot has not received a message during the travelled distance $S_{s}$ in the consensus process, the robot will terminate the consensus process. In this case, the decision is based on ${\hat{\pi }}_{e,i}\left(0\right)$.

## 6. Dynamic Swarm Case

The iterative estimate and consensus processes must be used alternatively to be able to adjust to changes in swarm density in real time. The robots will perform these processes at different speeds due to the randomness of the process completion time, though, when the robot instantly switches to the estimating process to estimate the new value as soon as the consensus process is complete. The opinions of the robots in the swarm may no longer be consistent if this procedure is out of phase among them.

**Figure 3:**
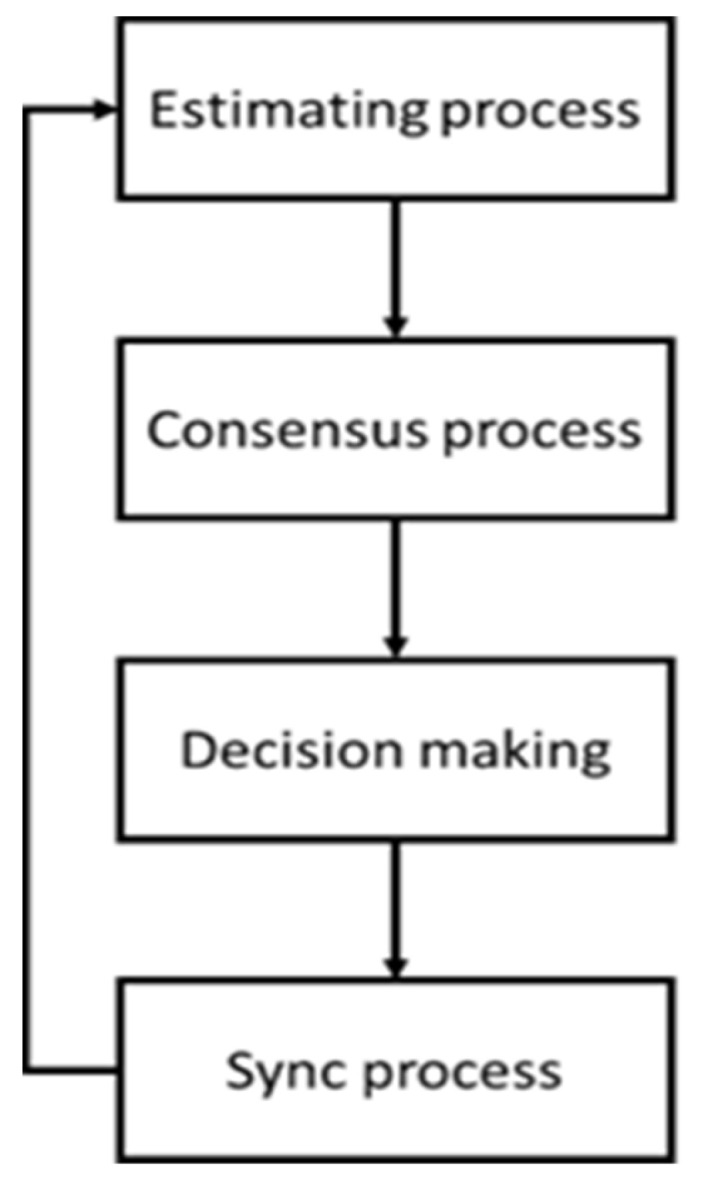
The whole process of proposed method.

After consensus is reached and before the entire process starts over again, an intermediate phase called synchronization is implemented in order to address this issue. The procedure helps robots that have completed the consensus process to wait for other robots that are still in the process of reaching the consensus, without synchronizing the clocks of the robots in the swarm. Figure 3 provides a summary of the full procedure for the swarm’s collective perception of density tailored to dynamic swarm.

As shown in Figure 3, the robot will transition to the sync procedure after completing the consensus process. The robot will keep track of the travelled distance $S_{w}$ during this operation. The sync procedure ends and the robot resumes to the estimating process if the counter reaches $S_{w}$. The robot will not automatically send any messages during the sync process. However, if it receives a message from another robot from the consensus phase, it will reply with its decision rather than an estimated value ${\hat{\pi }}_{e,i}$. The asymptotic average consensus is maintained in this manner. Robotic consensus processes can be considered completed when the correlation between the robot’s present estimated value and the intended value matches the majority decision the robot has received. The comparison between the estimated value at the time and the desired value will lead to the decision of the robots in this scenario.

## 7. Performance Evaluation

**Figure 4:**
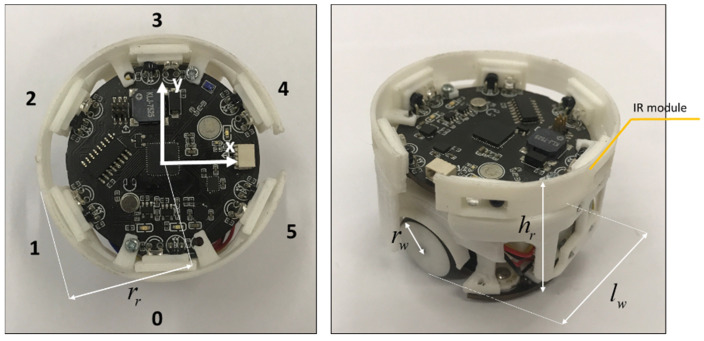
The robot platform utilized in this research is depicted in the image. On the left-hand side, there is a top view of the robot platform, including the IR-module with annotation numbers ranging from 0 to 5 that indicate their order and local frame notations. On the right-hand side, there is a side view of the robot platform.

The performances of the proposed model are evaluated according to the test which is conducted in a real experiment. All tests use the same robot platform which has a top and perspective view illustrated in Figure 4. The robot platform is cylindrical in shape with a radius $r_{r}$ of 25 mm and height $h_{r}$ of 40 mm, respectively. The inter-wheel distance $l_{w}$ and radius of each wheel $r_{w}$ are 40 mm and 10 mm, respectively. Given that the robot is equipped with 6 infrared modules, as shown in Figure 4, arranged in a specific order, the maximum number of neighbors of robot ${\left|F_{i}\right|}_{\max}$, $\forall i\in \left\{1,2,\ldots ,N\left(t\right)\right\}$ obviously is 6.

Without adding information, the default communication range and default sensing range $r_{s}$ of these infrared modules are approximately 180 mm. Finally, the robot’s linear speed can reach 35 mm/s. Due to the communication speed not being fast enough, the maximum linear speed has been set to 20 mm/s according to our experiments. The default initiation constants and parameters used in this section were listed in Table 1.

The test area for the swarm robots in this experiment is a square-shaped workspace measuring 2000 × 2000 mm^2^, marked by a black line to indicate the boundaries. Three infrared sensors are installed at the bottom of each robot to determine the boundaries of the work area. The message transmission process, which contains the message protocol and estimated value time of the robot, takes approximately 10 ms.

**Figure 5:**
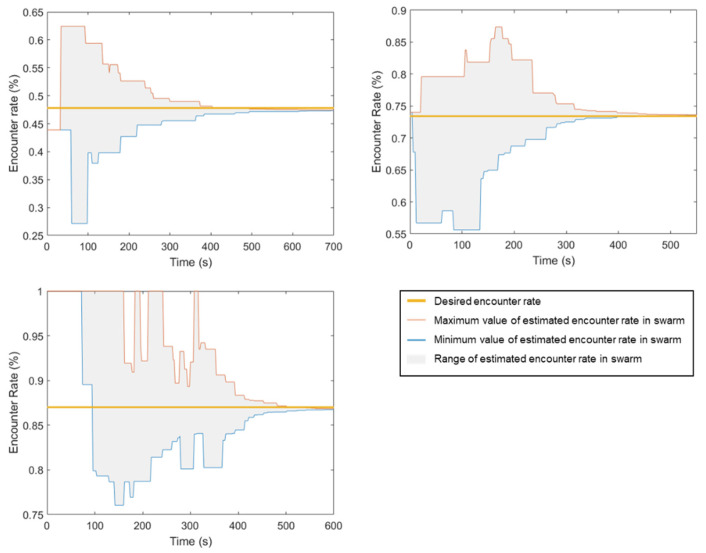
The results of experiments conducted to evaluate the convergence of the estimated encounter rate in the swarm are presented for a static swarm size scenario. The experiments were conducted for a swarm size of 20 (**top-left**), 40 (**top-right**), and 60 (**bottom-left**).

Since the swarm’s decision-making is based on the estimated encounter rate of the robots, the value that all robots converge to is crucial. Therefore, the convergence of estimated encounter rates of the swarm is first evaluated under the static swarm size. In this case, we set ϑ=0, i.e., the consensus process will continue to run until the experiment is terminated by the experimenter. Three experiments, in which the swarm size is set to 20, 40 and 60, are conducted to evaluate the convergence. According to Equations (11) and (16), the desired encounter rates $\pi_{e}\in \left\{0.4811,0.7339,0.865\right\}$ correspond to $N\in \left\{20,40,60\right\}$. In order to obtain the results, a central server was set up for the task of collecting data from all robots in the swarm. Each robot was equipped with a Wi-Fi communication system previously connected to the server. The robot will continuously stream its data to the server, and the type of data collected depends on the experiment. For this experiment, robots presenting in the workspace will continuously transmit their current estimated encounter rate to the server. The results of these experiments are plotted in Figure 5.

In Figure 5, at the initial time of all conducted experiments, the estimated encounter rate of the robots is unknown, so the time from the beginning to the appearance of the first complete estimated robot is not recorded. The values $\min({\hat{\pi }}_{e})$ and $\max({\hat{\pi }}_{e})$ are only recorded when any robot enters the consensus process; therefore, at the initial time on the graphs, $\min({\hat{\pi }}_{e})=\max({\hat{\pi }}_{e})$. However, soon after, as other robots join the consensus process, the range between the maximum and minimum estimated encounter rates begins to increase, leading to a rise in $range({\hat{\pi }}_{e})$. The consensus process gradually decreases $range({\hat{\pi }}_{e})$ until the values converge if t→∞. During the consensus process, not all communication between robots is successful, which can cause the swarm to deviate from the original mean value of the encounter rate. Specifically, in the case of $\pi_{e}=0.4811$, at t=650s, when $range({\hat{\pi }}_{e})<\vartheta$ satisfies the consensus process condition, then $0.4736\leq {\hat{\pi }}_{e,i}\leq 0.474,\;\forall R_{i}\in S$. However, this deviation is not significant enough to affect the collective decision of the swarm.

Based on the results of the experiments, it can be affirmed that the convergent rate of the encounter rate is dependent on the density of the swarm, but not in a linear fashion. In general, as the number of robots in the swarm increases, the number of transmissions required for achieving a consensus also tends to increase. This is because each robot needs to communicate its current state to its neighbors in order to reach a consensus, and the number of neighbors increases with the number of robots. As a result, the overall communication overhead increases. However, this relationship may not always be straightforward, as the nature of the coordination problem, communication protocol and other factors can also impact the number of transmissions required. In our case, since the workspace remains constant, the encounter rate increases as the swarm size increases. As a result, the consensus time does not depend linearly on the swarm density.

**Figure 6:**
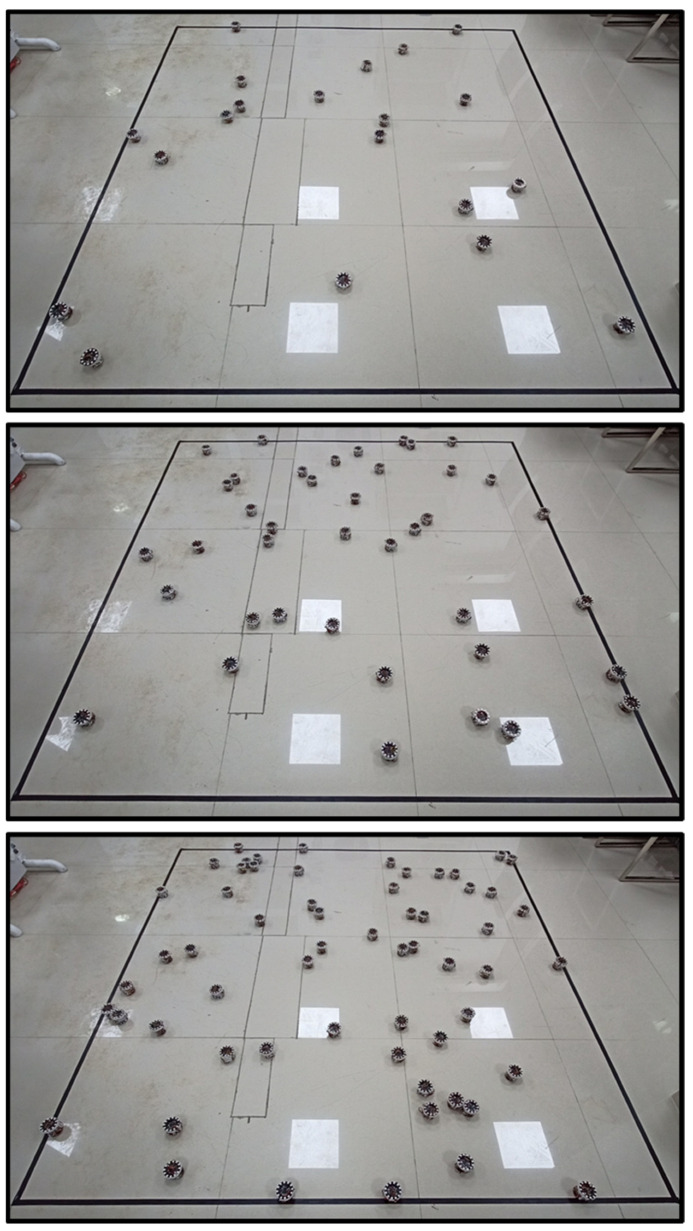
The snapshots of the second experiments used to evaluate the performance of the proposed method on swarms with varying sizes are shown. From top to bottom, the number of robots in the workspace are 20, 40 and 60, respectively.

To be able to demonstrate that the method is available for a swarm of robots with varying sizes, an experiment was performed on an initial swarm size $N\left(0\right)=20$, and a new robot was added every minute. The desired encounter rate $\pi_{e,d}$ is set to 0.8. The robot swarm is tasked with continuously making collective decisions about whether the current population of robots will satisfy a given desired encounter rate. The results of the experimental process are shown in two graphs: (1) $range({\hat{\pi }}_{e})$, $E({\hat{\pi }}_{e})$ and $\pi_{e}$ over time; (2) different number of robot decisions over time. Snapshots of this experiment are recorded and shown in Figure 6.

**Figure 7:**
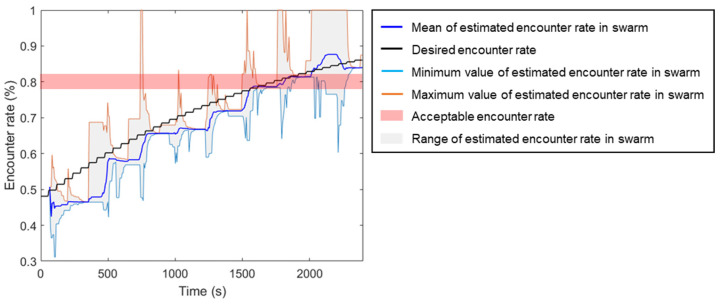
The recording of range of estimated encounter rate $range({\hat{\pi }}_{e})$, mean of estimated encounter rate $E({\hat{\pi }}_{e})$ and desired encounter rate $\pi_{e}$ over time during the experiment.

According to Figure 7, the mean of the estimated encounter rate over the swarm is underestimated compared to the actual encounter rate due to the latency of the proposed algorithm. When new robots are added, the desired encounter rate increases instantly, but the encounter rate estimation value for previous robots depends on the robot density in the past. These robots only update the new value when they complete the sync process and start a new estimation process. Therefore, if the change rate of the desired encounter rate increases at a higher rate, this requires the algorithm to respond faster; otherwise, the difference between the desired encounter and mean of the estimated encounter rate will increase significantly, resulting in the robot’s inability to make accurate decisions.

**Figure 8:**
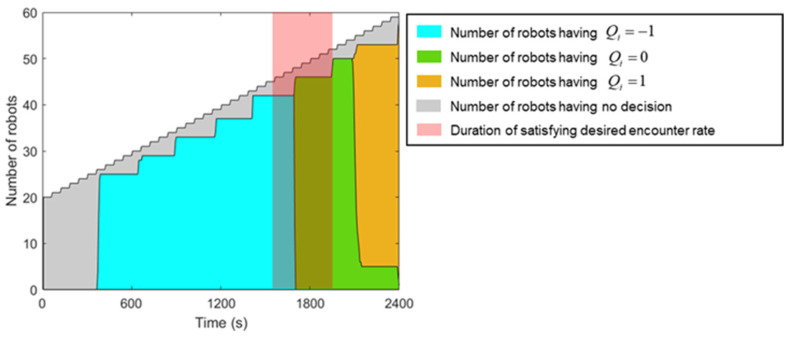
Number of robots at each decision and total number of robots in the swarm at each time point of the experiment.

In Figure 8, it can be observed that the consensus process, most of the time, enables the swarm to make a collective decision, except for the newly added robots that are still in the estimating process. Additionally, the sync process ensures that the robots return to the estimating process almost simultaneously. Consequently, the decision delay depends on the total time taken from when the robot starts estimating the encounter rate to the end of the sync process. The threshold error and the rate of change of the encounter rate must be selected to match the latency of the method. In this case, with σ=0.02 and the swarm growth rate as 1 robot/min, the duration of satisfying the desired encounter rate is about 350 s. This interval allows the method to respond in time, and all robots capable of making a decision at this time interval can make the appropriate decision.

## 8. Conclusions

In this study, a methodology that enables robot swarms to collectively assess whether their actual swarm density exceeds, is insufficient or is approximately comparable to a specified desired swarm density is introduced. The main goal of the proposed method is to enable the robots to collectively estimate the swarm density by assessing how frequently they interact with their neighbors, which will enable the swarm to come to a consensus. The proposed method does not require robots to have knowledge about their workspace, swarm size or their localization in the workspace. However, the trajectories of the robots in the swarm must be uniformly ergodic and the communication system must be a bidirectional communication with explicit messages. The experiments conducted on our proposed swarm robot platform show that the method is applicable to a dynamic swarm.

## Figures and Tables

**Table 1:** Constants and default parameters used in experiments.

Parameter	Description	Value
k	Number of samples which are used to estimate encounter rate in estimating process.	20
$S_{s}$	The maximum sample value in terms of travelled distance in estimating process.	5 m
$S_{q}$	The maximum duration in terms of travelled distance to self-broadcast current estimated encounter rate in consensus process.	1 m
$S_{w}$	The counter in terms of travelled distance in sync process.	10 m
ϑ	Acceptable range.	0.001
σ	Threshold error.	0.02

## Data Availability

Not applicable.
